# Enhanced immune activation within the tumor microenvironment and circulation of female high-risk melanoma patients and improved survival with adjuvant CTLA4 blockade compared to males

**DOI:** 10.1186/s12967-022-03450-3

**Published:** 2022-06-03

**Authors:** Mariam Saad, Sandra J. Lee, Aik Choon Tan, Issam M. El Naqa, F. Stephen Hodi, Lisa H. Butterfield, William A. LaFramboise, Walter Storkus, Arivarasan D. Karunamurthy, Jose Conejo-Garcia, Patrick Hwu, Howard Streicher, Vernon K. Sondak, John M. Kirkwood, Ahmad A. Tarhini

**Affiliations:** 1 Departments of Cutaneous Oncology and Immunology, H. Lee Moffitt Cancer Center and Research Institute, Tampa, FL 33612 USA 10920 McKinley Dr.,; 2 Dana Farber Cancer Institute and Harvard Medical School, Boston, MA USA; 3 Dana Farber Cancer Institute, Boston, MA USA; 4 Univ. California San Francisco and The Parker Institute for Cancer Immunotherapy, San Francisco, CA USA; 5 Allegheny Health Network Cancer Institute, Pathology, Pittsburgh, PA USA; 6 University of Pittsburgh School of Medicine (UPSOM), Pittsburgh, PA USA; 7 National Cancer Institute, Rockville, MD USA; 8 UPMC Hillman Cancer Center, University of Pittsburgh, Pittsburgh, PA USA; 9 Department of Biostatistics and Bioinformatics, H. Lee Moffitt Cancer Center and Research Institute, Tampa, USA Florida; 10 Department of Machine Learning, H. Lee Moffitt Cancer Center and Research Institute, Tampa, USA; 11 Department of Cutaneous Oncology, H. Lee Moffitt Cancer Center and Research Institute, Florida Tampa, USA; 12 Department of Immunology, H. Lee Moffitt Cancer Center and Research Institute, Florida Tampa, USA; 13 Administration, Cutaneous Oncology, Immunology, H. Lee Moffitt Cancer Center and Research Institute, Florida Tampa, USA

**Keywords:** Melanoma, Adjuvant, Female, Male, Ipilimumab, Interferon

## Abstract

**Background:**

We hypothesized that a gender difference in clinical response may exist to adjuvant CTLA4 blockade with ipilimumab versus high-dose IFNα (HDI). We investigated differences in candidate immune biomarkers in the circulation and tumor microenvironment (TME).

**Patients and methods:**

This gender-based analysis was nested within the E1609 trial that tested adjuvant therapy with ipilimumab 3 mg/kg (ipi3) and 10 mg/kg (ipi10) versus HDI in high risk resected melanoma. We investigated gender differences in treatment efficacy with ipi3 and ipi10 versus HDI while adjusting for age, stage, ECOG performance (PS), ulceration, primary tumor status and lymph node number. Forest plots were created to compare overall survival (OS) and relapse free survival (RFS) between ipi and HDI. Gene expression profiling (GEP) was performed on tumors of 718 (454 male, 264 female) patients. Similarly, serum and peripheral blood mononuclear cells (PBMC) samples were tested for soluble and cellular biomarkers (N = 321 patients; 109 female and 212 male).

**Results:**

The subgroups of female, stage IIIC, PS = 1, ulcerated primary, in-transit metastasis demonstrated significant improvement in RFS and/or OS with ipi3 versus HDI. Female gender was significant for both OS and RFS and was further explored. In the RFS comparison, a multivariate Cox regression model including significant variables indicated a significant interaction between gender and treatment (*P* = 0.024). In peripheral blood, percentages of CD3+ T cells (*P* = 0.024) and CD3+ CD4+ helper T cells (*P* = 0.0001) were higher in females compared to males. Trends toward higher circulating levels of IL1β (*P* = 0.07) and IL6 (*P* = 0.06) were also found in females. Males had higher percentages of monocytes (*P* = 0.03) with trends toward higher percentages of regulatory T cells (T-reg). Tumor GEP analysis supported enhanced infiltration with immune cells including gammadelta T cells (*P* = 0.005), NK cells (*P* = 0.01), dendritic cells (*P* = 0.01), CD4+ T cells (*P* = 0.03), CD8+ T cells (*P* = 0.03) and T-reg (*P* = 0.008) in the tumors of females compared to males and a higher T-effector and IFNγ gene signature score (*P* = 0.0244).

**Conclusion:**

Female gender was associated with adjuvant CTLA4 blockade clinical benefits and female patients were more likely to have evidence of type1 immune activation within the TME and the circulation.

*Trial registration* ClinicalTrials.gov NCT01274338. Registered 11 January 2011, https://www.clinicaltrials.gov/ct2/show/NCT01274338

**Supplementary Information:**

The online version contains supplementary material available at 10.1186/s12967-022-03450-3.

## Introduction

Melanoma accounts for the majority of skin cancer deaths in the United States. An estimated total of 7650 deaths will be attributed to melanoma in 2022 [1]. While early-stage resectable low-risk melanoma can be cured by surgical excision alone, later high-risk stages are managed with the postoperative addition of systemic adjuvant therapy that can reduce the risks of recurrence and death [2]. In 2015, ipilimumab 10 mg/kg (ipi10) received regulatory approval in the U.S. as the first immune checkpoint inhibitor (ICI) adjuvant therapy for high-risk resected melanoma, almost 10 years after the approval of adjuvant high-dose interferon-alpha (HDI) [3]. The North American Intergroup Phase III adjuvant trial E1609 tested ipilimumab 3 mg/kg (ipi3) versus HDI (primary comparison) or ipi10 versus HDI and demonstrated significant overall survival (OS) improvement with ipi3 versus HDI (hazard ratio [HR], 0.78; 95.6% repeated CI 0.61 to 0.99; *P* = 0.044) and no significant differences in survival between ipi10 and HDI [4]. Comparing ipi3 and ipi10, there were significant differences in toxicity rates in favor of ipi3 while recurrence and survival rates were similar.

While sexual dimorphism in immunity is acknowledged, sex-based responses to immunotherapies continue to be poorly understood [5]. Gender-based differences in cancer survival are well established in melanoma, with females having a significant survival advantage when compared to males. Cancer-specific survival differences in favor of females appear to decrease, however with increasing age [6], and with increasing metastatic tumor load [7]. While non-biological factors could be associated with this variance, such as a proposed more protective health-seeking behavior in women as well as improved reporting and access to health care, similar trends in cancer-related survival in favor of women were reported when these factors are accounted for [7]. Furthermore, sex hormonal differences have been hypothesized to differentially affect immune responses to immunotherapies [5]. Hormonal studies in murine models have demonstrated gender differences in melanoma outcomes that may be hormonally driven [9]. This is in addition to reported associations between the levels of estrogen and estrogen receptor expression in melanoma with patient survival in women [8]. When it comes to response to ICIs, gender-based differences have not been consistent in recent analyses of immunotherapy clinical trials. While one meta-analysis of recent immunotherapy clinical trials found significant gender-based differences in clinical benefits from ICIs in patients with metastatic melanoma [9], another metaanalysis found no significant association between gender and ICI survival benefits [10].

Therefore, there is a need to further investigate the contribution of sex to patient immunity and clinical benefits from ICIs in well-conducted randomized clinical trials such as E1609 with available biospecimens for correlative scientific testing. Here, based on our observations and literature reports we hypothesized that there is a gender difference in response to adjuvant immunotherapy with ipilimumab (ipi3 or ipi10) versus HDI as tested in the E1609 trial and investigated treatment efficacy between ipi and HDI in the subgroup of gender while controlling for other prognostic factors in a multivariate model. In addition, we hypothesized that male–female disparities in clinical benefits from ICIs are supported by differences in candidate immune biomarkers in the circulation and the tumor microenvironment (TME) of female and male patients.

## Patients and methods

### Patients

E1609 was a phase III study that enrolled patients with high-risk melanoma of cutaneous or unknown primary origin. Eligibility criteria included histological confirmation of melanoma. Patients were randomized and were rendered disease-free surgically within 12 weeks of randomization on the trial and were required to have AJCC 7th edition stages IIIB, IIIC, M1a or M1b [4]. Other criteria included ECOG performance status (PS) of 0 or 1 and passing screening safety laboratory testing criteria. Autoimmune disorders and conditions of immunosuppression that necessitated the use of systemic corticosteroids or other immunosuppressants were not permitted.

### Trial design and treatments

E1609 was an open-label phase III trial that randomized melanoma patients to systemic adjuvant therapy with ipi10, HDI or ipi3. Patients were stratified by the AJCC 7th edition stage groups of IIIB, IIIC, M1a and M1b [4]. Clinical trial design details and additional information related to the clinical trial endpoint points, treatment regimens, randomization specifics, and trial oversight were previously published [4]. Patient disposition is described in the consort diagram included in Additional file 1: Fig. S1. All patients provided an IRB-approved written informed consent.

### Methods and statistical analysis

E1609 demonstrated significant OS benefit with ipi3 versus HDI. We investigated treatment efficacy between ipi and HDI in the subgroups by gender (female, male), age (< 55 or ≥ 55), stage at study entry (AJCC 7th edition IIIB, IIIC, M1a/1b), ECOG performance status (PS 0, 1), primary tumor ulceration (yes, no), primary tumor identification (known, unknown), number of lymph nodes involved (0, 1, 2–3, 4 +). Forest plots were created to compare OS and RFS with ipi3 versus HDI and ipi10 versus HDI using the concurrently randomized ITT populations. For the estimated HRs, 95% confidence intervals were created for all subgroups. Univariate and Multivariate analyses were conducted with the multivariate Cox regression analysis used to adjust for confounders.

#### Gene expression profiling (GEP)

GEP was performed on the tumor biopsies of 718 (454 male, 264 female) melanoma patients. Only metastatic tumors were included that were resected to render patients disease free prior to clinical trial enrollment. Microdissection of Formalin-Fixed Paraffin-Embedded (FFPE) tumor specimens was performed manually using an inverted microscope (Nikon Eclipse TE200) as needed to obtain a minimum of 90% tumor cells for RNA purification. Dissection involved scraping cells from unstained sections of 5 micron thickness on slides aligned in register with serially cut hematoxylin and eosin stained specimens including tumor domains demarcated by a surgical pathologist (A. K.). RNA purification was performed using the Qiagen miRNeasy FFPE Kit and protocol (Qiagen, Valencia, CA) with isolated RNA suspended in nuclease-free water. Inclusion in subsequent in vitro amplification (IVT) assays was determined both by spectrophotometric absorption ratio [260/280 > 1.8 (NanoDrop, Wilmington, DE)] and RIN values (RNA Integrity Index) determined via microchip electrophoretic analysis (Agilent Bioanalyzer 2100, Agilent Technologies, Santa Clara, CA). We previously established that RIN values ranging from 5.0 to 8.0 in RNA from FFPE specimens can undergo successful in vitro transcription and amplification using a multiplex primer approach. Amplification was performed using the NuGen whole transcription method comprising the Ovation FFPE WTA assay (NuGEN, San Carlos, CA) employing random and 3′ primers to eliminate amplification bias beginning with 100 ng total RNA. Confirmation of cDNA diversity was obtained using the Bioanalyzer 2100 to generate an electrophoretogram for each amplification reaction regarding sample yield, integrity and size diversity compared to a laboratory human RNA standard and a Universal Human Reference RNA (Stratagene, La Jolla, CA). 5 µg of purified cDNA were incubated with fragmentation buffer (NuGEN, San Carlos, CA) at 37 °C for 30 min, then 95 °C for 2 min. The cDNA samples were hybridized on Affymetrix GeneChip HG U133A 2.0 arrays which comprehensively represent the functionally characterized human genome with overlapping probe sets for transcripts.

#### Data analysis of gene expression profiles

Robust Multi-array Average (RMA) method was used to normalize raw microarray data as previously described [11, 12]. Genes with multiple probe sets were collapsed by using the probe with maximum gene expression. Gene set enrichment analysis (GSEA) was performed by comparing the female and male tumor samples [13]. In this study, KEGG pathways gene sets were obtained from MSigDB to interrogate the enrichment of pathways in the female and male samples [14]. In order to further deconvolute the cell types in the bulk transcriptomics, we used gene sets obtained from CIBERSORT [15, 16], and TIMEx [17], in comparing the female versus male samples. Gene sets with a false discovery rate (FDR) q-value < 0.15 were deemed as significant. We also tested previously published prognostic gene signatures of immunotheraphy in comparing female versus male tumors including IFNγ 6-gene signature (IDO1, CXCL10, CXCL9, HLA-DRA, IFNG, STAT1) [18], and T-effector and IFNγ gene signature (CD8A, GZMA, GZMB, IFNG, EOMES, CXCL9, CXCL10, TBX21) [19]. For each sample, we computed a gene signature score by averaging the standardized z-score for the genes in the signature. For each of these gene signatures [20]. Mann–Whitney U test was performed by comparing female and male and p < 0.05 was deemed as statistically significant.

#### Serum and peripheral blood mononuclear cells (PBMC) data analysis

Peripheral blood samples were tested for soluble (Luminex) and cellular (multicolor flow cytometry) prognostic biomarkers in a subset of patients (N = 321; 109 female and 212 male). Mann–Whitney U test was performed by comparing between female and male and p < 0.05 was deemed as statistically significant.

#### Peripheral blood

Red top vacutainer tubes (BD, no anticoagulant) were used for serum collection and all samples were processed within 24 h of collection (samples received before 5 pm were processed upon receipt, those arriving after 5 pm were processed the following morning). Serum samples were centrifuged at 2500 rpm for 10 min at 4 °C according to laboratory standard operating procedures (SOPs) and single use aliquots of each patient’s sera were then stored at − 80 °C. For PBMC, blood was drawn into heparin tubes and processed by the Immunologic Monitoring Laboratory upon receipt. PBMCs were obtained from the blood samples by ficol density-gradient centrifugation and stored frozen. The laboratory freezers were monitored continuously for any temperature fluctuations and maintained the samples at -80 °C.

#### Multiplex serum cytokine analysis

21 serum cytokines were selected for analysis based on function. These included Th1 type cytokines (IL-12p70, IL-17, IL-2, IP-10), proinflammatory (IL-1α, IL-1β, IL-6, TNF-α, TNF-RII, IL-2R, IL-8, CRP, IL-17, IFN-α), immunoregulatory (TGF-α, IL-10, TIMP1), growth factor (VEGF-A), and other/chemokines (CCL3/MIP-1α, CCL4/MIP-1β, CXCL9/MIG, CXCL11/I-TAC). The xMAP Luminex serum assay for these cytokines was performed according to the manufacturer’s protocol (BioSource International (Camarillo, CA) as previously described [21], and laboratory SOPs, and analyzed on the Bio-Plex suspension array system (Bio-Rad Laboratories, Hercules, CA). Experimental data was analyzed using five-parametric curve fitting and assay controls included kit standards and multiplex QC controls (R & D Systems). Inter-assay variabilities for individual cytokines were 1.0 to 9.8% and intra-assay variabilities were 3.6 to 12.6% (information provided by Biosource International and validation performed in our laboratory). C-reactive Protein (CRP) was run singly as it requires different dilutions.

### Multicolor flow cytometry

Multicolor flow cytometry was used to compare cell subset phenotypes on thawed patient peripheral blood mononuclear cells (PBMC), with healthy donor controls, run according to laboratory SOPs. Regulatory T cells (Treg) were defined as CD4+ CD25+ FOXP3+ or CD4+ CD25hi+ CD39+ cells, to incorporate the candidate functional marker CD39 as previously described [22]. Myeloid-derived-suppressor cells (MDSC) were defined as cells expressing Lin-neg/HLA-DR−/CD33+/CD11b+ in either a “lymphocyte” (small FSCxSSC) gate, or in a “monocyte” (larger FSCxSSC) gate, and as HLA-DR+/lo CD14+ cells in a large gate as previoulsy described [22]. We also tested the frequencies of CD4+ and CD8+ T cells specific to shared tumor-associated antigens (gp100, MART-1, NY-ESO-1) utilizing overlapping peptide libraries (15-mer peptides overlapping by 4) and a short (4–5 h) in vitro culture to identify activated (CD69+) and cytokine producing (intracellular IFNγ+) T cells. Detailed methods were described previously [22].

## Results

The characteristics of patients enrolled in E1609 and the treatment details as well as the incidence rate of irAEs were previously published [4]. Table 1 summarizes the baseline and disease characteristics of the E1609 study population included in this analysis.

**Table 1:** Patient demographics and baseline disease characteristics

	Ipilimumab 10 mg/kg (ipi10) (n = 511)	HDI (n = 636)	Ipilimumab 3 mg/kg (ipi3) (n = 523)
Age Median (range)	52 years (18–80)	54 years (18–83)	54 years (19–80)
Stage (AJCC7)			
IIIB	268 (52.5%)	331 (52.0%)	280 (53.5%)
IIIC	205 (40.1%)	253 (39.8%)	205 (39.2%)
M1a	28(5.5%)	34 (5.4%)	28 (5.4%)
M1b	10 (1.9%)	18(2.8%)	10 (1.9%)
Sex			
Male	342 (66.9%)	395 (62.1%)	328 (62.7%)
Female	169 (33.1%)	241 (37.9%)	195 (37.3%)
PS			
0	426 (83.5%)	533 (83.8%)	439 (84.7%)
1	85(16.5%)	102 (16.0%)	82 (15%)
Unknown/Missing	0	1 (.2%)	2 (.3%)
Primary tumor status			
Unknown	56 (11.0%)	103 (16.2%)	84 (16.1%)
Ulceration			
No	216 (42.3%)	263 (41.4%)	187 (35.4%)
Yes	227 (44.4%)	260 (41.5%)	252 (46.9%)
Unknown (most due to unknown primary)	68 (13.3%)	113 (18.1%)	84 (17.7%)
Microscopic LN Involvement			
Yes (among IIIB/IIIC)	233 (49.2%)	285 (50.5%)	247 (50.9%)

**Fig. 1:**
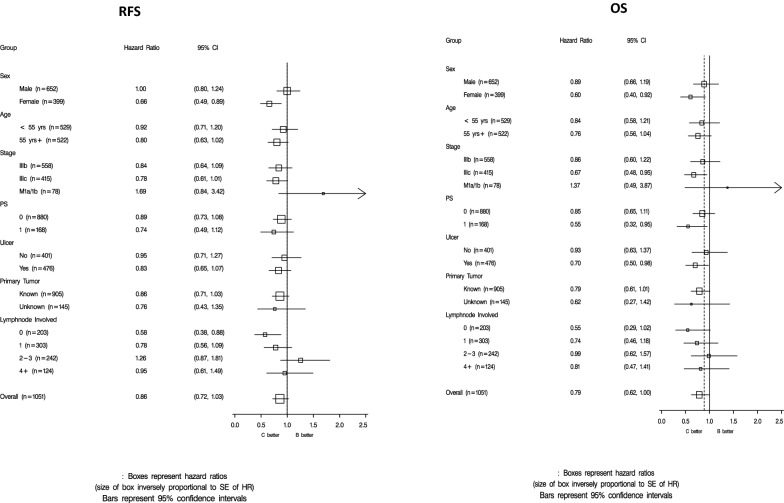
Forest plots comparing relapse free survival (RFS) and overall survival (OS) for ipilimumab 3 mg/kg versus high dose interferon-alfa

Using the concurrently randomized ITT populations in the subgroup analyses, forest plots were created to compare ipi3 versus HDI in terms of RFS and OS (Fig. 1) and to compare ipi10 versus HDI in terms of RFS and OS (Additional file 2: Fig. S2).

In investigating RFS with ipi3 versus HDI, the subgroups of female, stage IIIC, PS = 1, ulcerated, in-transit without lymph node involvement demonstrated significant improvement in OS and/or RFS with ipi3 versus HDI as summarized in Table 2. Female gender was significant for both OS and RFS and was further explored. A multivariate Cox regression model including gender, treatment and interaction term of gender*treatment, indicated a significant interaction between gender and treatment (*P* = 0.026). Including gender, PS (0 versus 1), age (< 55 versus ≥ 55), ulceration (yes versus no), stage (IIIB, IIIC, M1a, M1b), treatment and interaction term of gender*treatment, indicated a significant interaction between gender and treatment (*P* = 0.024).

**Table 2:** Treatment efficacy between ipi3 and HDI by subgroup

Group	HR, 95% CI	
	OS	RFS
Female gender	0.60 (0.40, 0.92)	0.66 (0.49, 0.89)
In-transit, LN-ve	0.55 (0.29, 1.02)	0.58 (0.38, 0.88)
Ulceration	0.70 (0.50, 0.98)	0.83 (0.65, 1.07)
Stage IIIC	0.67 (0.48, 0.95)	0.78 (0.61, 1.01)
PS = 1	0.55 (0.32, 0.95)	0.74 (0.49, 1.12)

Estimated hazard ratios (HR) with 95% confidence intervals (CI) are provided. The subgroups of female, stage IIIC, PS = 1, ulcerated, in-transit without lymph node involvement demonstrated significant improvement in OS and/or RFS with ipi3 versus HDI. Female gender was significant for both OS and RFS

When exploring age further in the univariate analyses in the ipi3 versus HDI comparison, older women appeared to drive most of the difference (age ≥ 55: OS, *P* = 0.02 and RFS, *P* = 0.08; differences non-significant for age < 55).

While similar trends were clearly seen, no significant interactions between gender and treatment effect were found in the OS multivariate analysis for ipi3 versus HDI or in the comparison of ipi10 versus HDI.

**Fig. 2:**
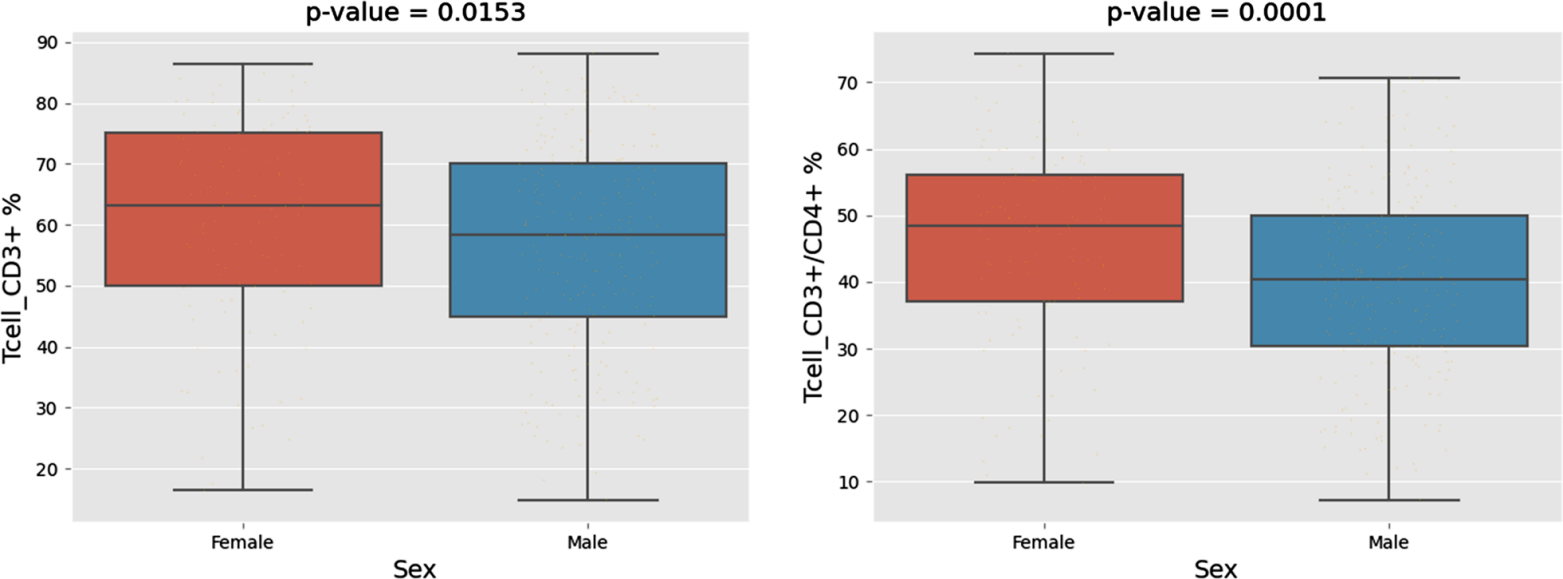
Multicolor flow cytometry of peripheral blood mononuclear cells (PBMCs). The percentages of CD3+ T cells (*P* = 0.04) and CD3+ CD4+ helper T cells (*P* = 0.001) were significantly higher in female patients compared to males

**Fig. 3:**
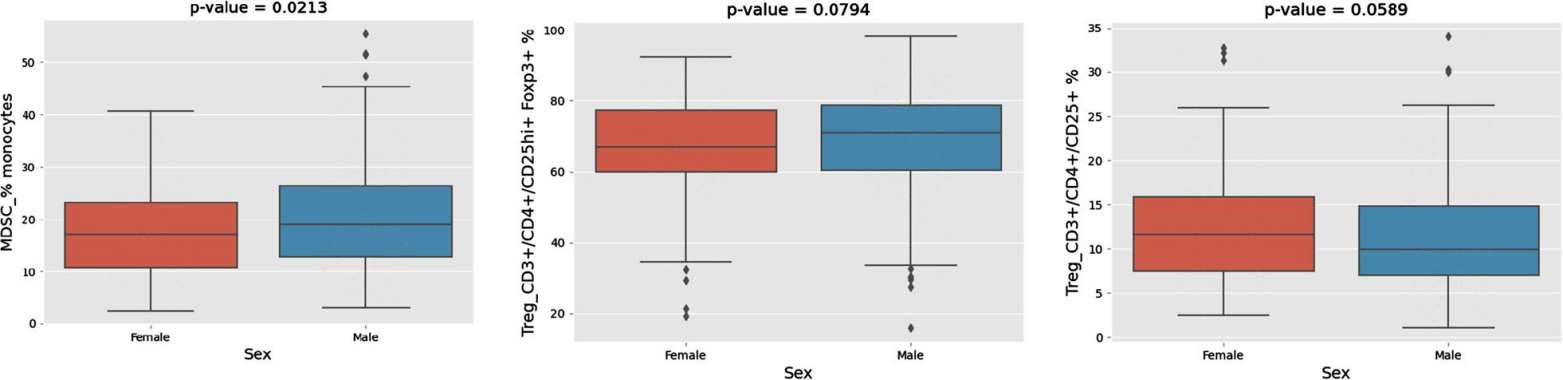
Multicolor flow cytometry of peripheral blood mononuclear cells (PBMCs). Significantly higher percentages of monocytes (P = 0.03) and trends toward higher percentages of CD3+/CD4+/CD25hi+/Foxp3+ (P = 0.1) and CD3+/CD4+/CD25+/CD127low+ (P = 0.1) T-reg in male patients compared to females

Among the subset of patients (N = 321) tested for circulating biomarkers, females were significantly younger than males (*P* = 0.03). Testing PBMCs, the percentages of CD3+ T cells (*P* = 0.04) and CD3+ CD4+ helper T cells (*P* = 0.001) were significantly higher in female patients compared to males (Fig. 2). Also, there were trends toward higher levels of proinflammatory cytokines IL-1β (*P* = 0.07) and IL6 (*P* = 0.06) in females (Additional file 3: Fig. S3). Conversely, males had significantly higher percentages of circulating monocytes (*P* = 0.03). Importantly, there were trends toward higher percentages of CD3+/CD4+/CD25hi+/Foxp3+ (*P* = 0.1) and CD3+/CD4+/CD25+/CD127low+ (*P* = 0.1) T-reg in male patients compared to females (Fig. 3).

Among the cohort of patients (N = 718) with tumor GEP data, females were significantly younger than males (*P* = 0.0009, U-test). As expected, when comparing the differentially expressed genes and pathways between female and male patient tumors, the top ranked genes were related to the sex chromosomes. To investigate the underlying immunologic differences related to female and male in response to immunotherapy, we performed GSEA using CIBERSORT gene signatures which are related to immune cell infiltration and activation. Interestingly, female patients’ tumors were significantly enriched in immune related pathways and genes compared to the tumors of male patients, with estimated enhanced immune cell infiltration including CD4+ T cells, CD8+ T cells, γδ T cells, NK cells, dendritic cells, Tregs and M1 macrophages (Table 3). Furthermore, we performed TIMEx analysis and male patients’ tumors were estimated to be enriched in tumor stromal endothelial cells as compared to female patients’ tumors (p = 0.0429, U-test).

**Table 3:** Immune related pathways found to be significantly enriched in the tumors of female patients compared to tumors of male patients as computed by gene set enrichment analysis (GSEA; utilizing CIBERSORT gene sets) (NES: Normalized enrichment score)

Name	NES	p-val	FDR q-val
T_CELLS_GAMMA_DELTA	2.16	0.0052	0.1141
NK_CELLS_ACTIVATED	1.97	0.0102	0.0607
NK_CELLS_RESTING	1.77	0.0118	0.0552
DENDRITIC_CELLS_ACTIVATED	1.73	0.0106	0.0465
T_CELLS_REGULATORY_(TREGS)	1.65	0.0085	0.0503
DENDRITIC_CELLS_RESTING	1.59	0.0140	0.0636
T_CELLS_CD8	1.52	0.0270	0.0830
MACROPHAGES_M1	1.50	0.0164	0.0824
T_CELLS_CD4_NAIVE	1.46	0.0294	0.0932

**Fig. 4:**
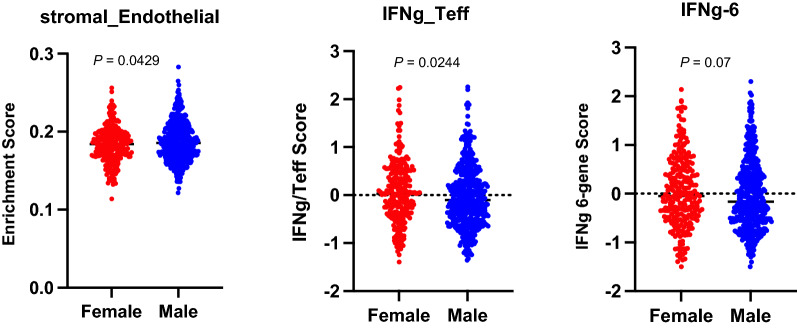
Gene expression changes in female versus male patients. T-effector and IFNγ gene signature was higher in female tumors as compared to male tumors (*P* = 0.0244) There was a trend towards a higher score for the IFNγ 6-gene signature in favor of female (*P* = 0.07). On the otherhand, endothelial cells were estimated to be enriched in the tumors of male patients as estimated by TIMEx (P = 0.0429)

To further explore gender-related differences in response to adjuvant immunotherapy, we evaluated published gene signatures that may be associated with immunotherapy benefits in female versus male tumors in this study. The T-effector and IFNγ gene signature was found to have a higher score in female tumors as compared to male tumors (*P* = 0.0244, U-test) and there was a trend toward a higher score for the IFNγ 6-gene signature in favor of female tumors (*P* = 0.07, U-test) (Fig. 4).

## Discussion

When exploring differences in gender response to immune-checkpoint inhibitors, results from the E1609 trial suggest superior clinical benefits from CTLA4 blockade in the subgroup of female patients. Particularly when accounting for potential confounders, females were shown to have significantly higher relapse-free survival rate as compared to males in the study comparison of ipi3 versus HDI with similar trends observed in the OS comparison and in investigating RFS and OS with ipi10 versus HDI. Overall, in melanoma, female patients have generally been reported to have improved survival including a lower risk of regional and systemic disease progression, and a higher likelihood of survival following disease progression [7, 23]. While the results of this study support the gender disparity in terms of benefit from adjuvant CTLA4 blockade, analyses of other ICI trials have not been consistent. Yang et al. reported no significant differences in gender-based benefits from ICIs therapy in a meta-analysis of 37 randomized clinical trials involving patients with advanced malignancies including melanoma [10]. On the other hand, Conforti et al. conducted a meta-analysis of 20 randomized trials of ICIs in advanced cancers including melanoma and suggested that men derived more survival benefits compared to women [9]. Overall, these meta-analyses constituted pan-cancer analyses, the number of men included was significantly higher than the number of women and the control arm in the melanoma studies was most often another ICI. Furthermore, the aforementioned meta-analyses included studies of inoperable metastatic disease rather than patients in the operable adjuvant setting which is the case of E1609.

Our clinical findings of potential improvement in survival for females following ipilimumab adjuvant therapy were also supported by our immune monitoring studies. In testing candidate circulating biomarkers females were shown to have significantly higher percentages of CD3+ T cells and CD3+ CD4+ helper T cells in addition to trends towards higher levels of proinflammatory cytokines IL1β and IL6 in females. Males had significantly higher percentages of monocytes with trends of higher percentages of CD3+/CD4+/CD25hi+/Foxp3+ and CD3+/CD4+/CD25+/CD127low+ regulatory T cells. Our findings are consistent with other immune monitoring reports in the literature of higher baseline numbers of CD3+ CD4+ helper T cells, a higher CD4+/CD8+ cell ratio in women compared to men [24], and lower regulatory T cell percentage in females than in males [25]. The monocyte frequency is a new finding and should be investigated further for the M1/M2 profile of those cells, and absolute counts in addition to frequencies. Because ipilimumab blockade of CTL4 induces immune-mediated tumoricidal actions by fortifying effector T cell activation [26], our findings support potential more pronounced immune effects with CTLA4 blockade in the peripheral blood of females as compared to males.

Immune escape in melanoma includes, and certainly is not limited to, altering immune cell functions such as impairing NK cell cytolytic activity, reducing stimulatory effects of dendritic cells upon effector T cells, promoting cytotoxic T cell anergy and stimulating Treg [27]. This is in addition to the ability of the tumor cells themselves to directly evade T cell surveillance and destruction. The nature of the TME transcriptome provides important clues that reflect the immunogenicity of the TME and its susceptibility to immunotherapy interventions. In this study we identified pathways and genes related to immune cell infiltration and activation that were significantly enriched in the tumors of females compared to males. We estimated enhanced immune cell infiltration in female tumors including CD4+ T cells, CD8+ T cells, γδT cells, NK cells and dendritic cells that support a more immunogenic TME and are prognostic or improved survival [28]. Similarly, the T-effector and IFNγ gene signature was found to have a higher score in female tumors and there was a trend towards a higher score for the of IFNγ 6-gene signature in favor of female tumors. These gene expression profiles were shown to be prognostic of improved survival in patients treated with ICIs [18, 19]. Our findings that these signatures were more pronounced in the tumors of females support our original hypothesis of a higher susciptibility to ICI induced immune responses in females with high-risk resected melanoma. Interestingly, we observed that genes related to stromal endothelial cells were significantly more expressed in the tumors of males. Cancer growth and metastasis are regulated in part by stromal cells such as fibroblasts and endothelial cells that imacpt the immune cell repertoire within the TME. Increased endothelial cell density may reflect a more angiogenic tumor where neoangiogenesis is a recognized hallmark of cancer that drives cancer progression and growth and confers poorer prognosis [29–31]. In terms of the tumor types tested and potential differences between males and females, all tumors were metastases. The types of metastases were cutaneous/subcutaneous, nodal or lung metastasis as reflected by the patients’ stage groups (IIIB, IIIC, M1a or M1b). We analyzed our cohort of 718 patients and have found no significant differences between females and males in terms of stage groups. Therefore, it is unlikely that there are significant differences between females and males in terms of the types of tumor tissue samples analyzed that may explain the difference in endothelial cell density.

Finally, we observed that females were significantly younger than males in our cohorts. This observation is consistent with the incidence of melanoma in the general population. Furthermore, in our investigation of gender differences in treatment efficacy we adjusted for age and other prognostic factors including stage, ECOG PS, ulceration, primary tumor status and lymph node number. In addition, when exploring age further in the univariate analyses in the ipi3 versus HDI comparison, older women appeared to drive most of the differences in survival (age ≥ 55: OS, P = 0.02 and a trend in RFS, P = 0.08; differences were non-significant for age < 55). Therefore, it is unlikely that the younger age of females is a major contributing factor to the outcomes seen in our analysis.

## Conclusions

Female gender was associated with adjuvant immunotherapeutic benefit and female patients were more likely to have evidence of immune activation within the TME and the circulation, supporting a potentially important role for factors related to female gender in the immune response against melanoma. These warrant further investigation.

## Supplementary Information

**Additional file 1: Figure S1.** E1609 Consolidated Standards of Reporting Trials diagram (adult patient populationa). aE1609 included a pediatric component (ages 12–17 years) consisting of three separate cohorts randomized to the three treatment regimens and analyzed separately for safety per study protocol. Total pediatric accrual was three subjects; bthese overlap with but are not limited to treatment-related grade 5 events previously reported; cconcurrently randomized cases. HDI, high-dose interferon alpha-2b; Ipi3, ipilimumab 3 mg/kg; Ipi10, ipilimumab 10 mg/kg; ITT, Intent to Treat.

**Additional file 2: Figure S2.** Forest plots comparing relapse free survival (RFS) and overall survival (OS) for ipilimumab 10 mg/kg versus high dose interferon-alfa.

**Additional file 3: Figure S3.** Serum cytokine analysis utilizing the xMAP Luminex serum assay. Trends toward higher levels of proinflammatory cytokines IL1beta (P = 0.07) and IL6 (P = 0.06) in females compared to males.

## Data Availability

The data sets generated, analyzed and reported in the present paper will be made available in the NCTN/NCORP Data Archive (https://nctn-data-archive.nci.nih.gov).

## Abbreviations

AJCC: American Joint Committee on Cancer
CTLA-4: Cytotoxic T lymphocyte antigen 4
ECOG-ACRIN: Eastern Cooperative Oncology Group-American College of Radiology Imaging Network
FFPE: Formalin fixed paraffin embedded
GSEA: Gene set enrichment analysis
GI: Gastrointestinal
MHC: Major histocompatibility complex
HDI: High-dose interferon-α
HR: Hazard ratio
ICI: Immune checkpoint inhibitor
irAE: Immune-related adverse event
Ipi3: Ipilimumab at 3 mg/kg
Ipi10: Ipilimumab at 10 mg/kg
KEGG: Kyoto Encyclopedia of Genes and Genomes
MUP: Melanoma of unknown primary
NES: Normalized enrichment score
PBMC: Peripheral blood mononuclear cells
PD-1: Programmed cell death protein 1
PS: Performance status
OS: Overall survival
RFS: Relapse free survival
TME: Tumor microenvironment
