# Supplementary figures and images for: Enhanced immune activation within the tumor microenvironment and circulation of female high-risk melanoma patients and improved survival with adjuvant CTLA4 blockade compared to males
Source: J Transl Med. 2022 Jun 3;20:253. doi: 10.1186/s12967-022-03450-3 (PMC9164320; doi:10.1186/s12967-022-03450-3)

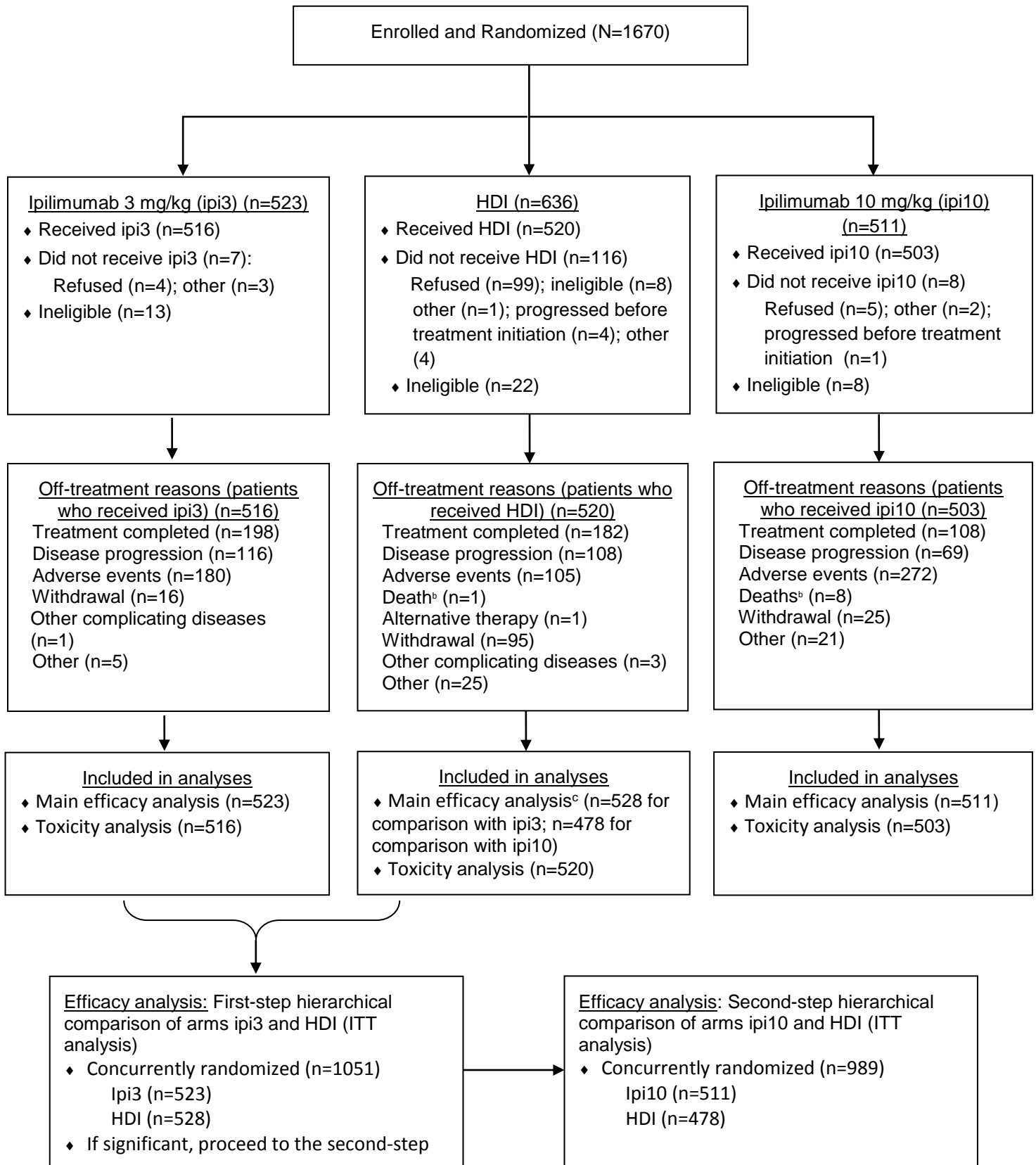

Supplement: Supplementary file 1 — Additional file 1: Figure S1. E1609 Consolidated Standards of Reporting Trials diagram (adult patient populationa). aE1609 included a pediatric component (ages 12–17 years) consisting of three separate cohorts randomized to the three treatment regimens and analyzed separately for safety per study protocol. Total pediatric accrual was three subjects; bthese overlap with but are not limited to treatment-related grade 5 events previously reported; cconcurrently randomized cases. HDI, high-dose interferon alpha-2b; Ipi3, ipilimumab 3 mg/kg; Ipi10, ipilimumab 10 mg/kg; ITT, Intent to Treat. [file 12967_2022_3450_MOESM1_ESM.pdf]
