# Supplementary material for: Enhanced immune activation within the tumor microenvironment and circulation of female high-risk melanoma patients and improved survival with adjuvant CTLA4 blockade compared to males
Source: J Transl Med. 2022 Jun 3;20:253. doi: 10.1186/s12967-022-03450-3 (PMC9164320; doi:10.1186/s12967-022-03450-3)
Supplement: Supplementary file 2 — Additional file 2: Figure S2. Forest plots comparing relapse free survival (RFS) and overall survival (OS) for ipilimumab 10 mg/kg versus high dose interferon-alfa. [file 12967_2022_3450_MOESM2_ESM.pdf]

Figure S2. Forest plots comparing relapse free survival (RFS) and overall survival (OS) for ipilimumab 10 mg/kg versus high dose interferon-alfa.

RFS

Group	Hazard Ratio	95% CI
Sex		
Male (n=647)	0.89	(0.71, 1.12)
Female (n=342)	0.79	(0.59, 1.06)
Age		
< 55 yrs (n=497)	0.91	(0.70, 1.19)
55 yrs+ (n=492)	0.78	(0.61, 0.99)
Stage		
IIIb (n=510)	0.89	(0.68, 1.16)
IIIc (n=399)	0.78	(0.60, 1.02)
M1a/1b (n=80)	1.09	(0.56, 2.10)
PS		
0 (n=815)	0.84	(0.69, 1.03)
1 (n=163)	0.87	(0.58, 1.32)
Ulcer		
No (n=416)	0.88	(0.66, 1.17)
Yes (n=424)	0.81	(0.62, 1.06)
Primary Tumor		
Known (n=862)	0.84	(0.69, 1.01)
Unknown (n=127)	0.87	(0.51, 1.50)
Lymphnode Involved		
0 (n=192)	0.76	(0.51, 1.14)
1 (n=299)	0.86	(0.61, 1.21)
2−3 (n=214)	1.12	(0.76, 1.66)
4+ (n=113)	0.87	(0.55, 1.40)
Overall (n=989)	0.85	(0.71, 1.01)

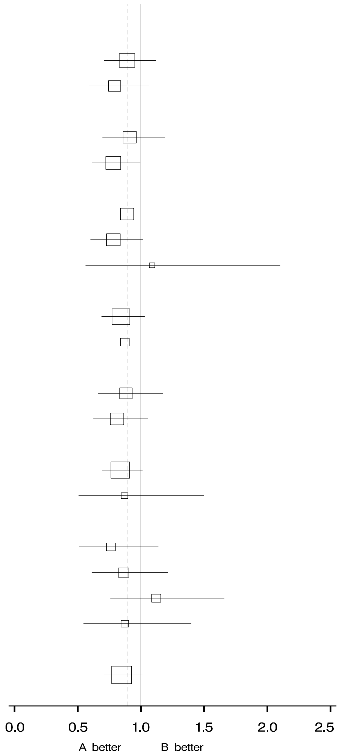

: Boxes represent hazard ratios
(size of box inversely proportional to SE of HR)
Bars represent 95% confidence intervals

OS

Group	Hazard Ratio	95% CI
Sex		
Male (n=647)	0.88	(0.66, 1.17)
Female (n=342)	0.86	(0.59, 1.26)
Age		
< 55 yrs (n=497)	0.95	(0.67, 1.34)
55 yrs+ (n=492)	0.80	(0.59, 1.09)
Stage		
IIIb (n=510)	0.93	(0.66, 1.31)
IIIc (n=399)	0.78	(0.56, 1.08)
M1a/1b (n=80)	1.38	(0.57, 3.39)
PS		
0 (n=815)	0.86	(0.66, 1.11)
1 (n=163)	0.91	(0.55, 1.50)
Ulcer		
No (n=416)	0.83	(0.58, 1.21)
Yes (n=424)	0.81	(0.58, 1.12)
Primary Tumor		
Known (n=862)	0.82	(0.65, 1.05)
Unknown (n=127)	1.18	(0.57, 2.44)
Lymphnode Involved		
0 (n=192)	0.73	(0.42, 1.27)
1 (n=299)	0.97	(0.63, 1.50)
2−3 (n=214)	1.07	(0.66, 1.74)
4+ (n=113)	0.89	(0.52, 1.53)
Overall (n=989)	0.87	(0.69, 1.09)

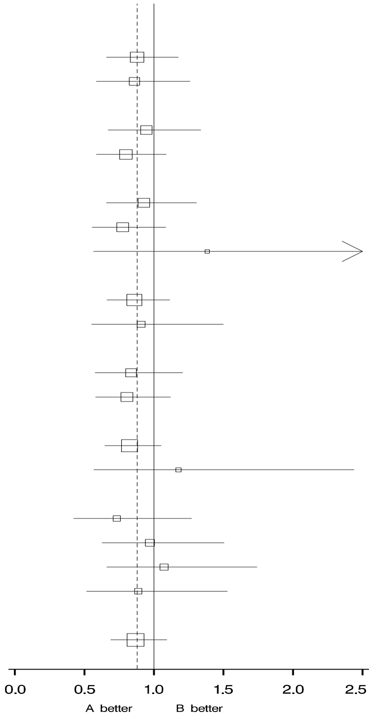

: Boxes represent hazard ratios
(size of box inversely proportional to SE of HR)
Bars represent 95% confidence intervals
