# Supplementary material for: Enhanced immune activation within the tumor microenvironment and circulation of female high-risk melanoma patients and improved survival with adjuvant CTLA4 blockade compared to males
Source: J Transl Med. 2022 Jun 3;20:253. doi: 10.1186/s12967-022-03450-3 (PMC9164320; doi:10.1186/s12967-022-03450-3)

Figure S3. Serum cytokine analysis utilizing the xMAP Luminex serum assay. Trends toward higher levels of proinflammatory cytokines IL1β (P=0.07) and IL6 (P=0.06) in females compared to males

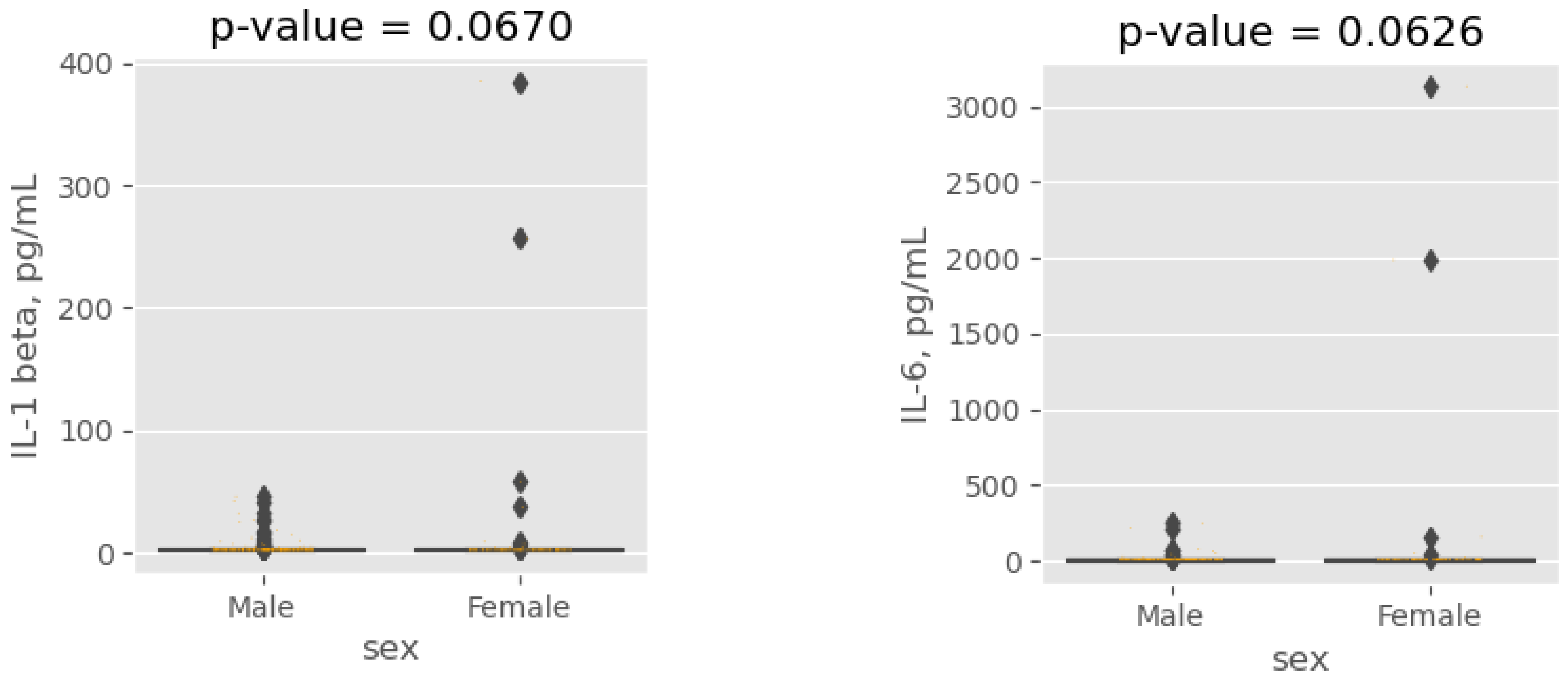

Supplement: Supplementary file 3 — Additional file 3: Figure S3. Serum cytokine analysis utilizing the xMAP Luminex serum assay. Trends toward higher levels of proinflammatory cytokines IL1beta (P = 0.07) and IL6 (P = 0.06) in females compared to males. [file 12967_2022_3450_MOESM3_ESM.pdf]
